# Transcription-associated metabolomic profiling reveals the critical role of frost tolerance in wheat

**DOI:** 10.1186/s12870-022-03718-2

**Published:** 2022-07-11

**Authors:** Liangjie Lv, Ce Dong, Yuping Liu, Aiju Zhao, Yelun Zhang, Hui Li, Xiyong Chen

**Affiliations:** 1 Institute of Cereal and Oil Crops, Hebei Academy of Agriculture and Forestry Sciences, Crop Genetics and Breeding Laboratory of Hebei, Shijiazhuang, 050000 China; 2 Handan Academy of Agricultural Sciences, Handan, 056000 Hebei China

**Keywords:** Wheat (*Triticum aestivum*), Frost-resistant, Transcriptome, Metabolome, Flavonoid biosynthesis, Sucrose and amino acid biosynthesis

## Abstract

**Background:**

Low temperature is a crucial stress factor of wheat (*Triticum aestivum L.*) and adversely impacts on plant growth and grain yield. Multi-million tons of grain production are lost annually because crops lack the resistance to survive in winter. Particularlly, winter wheat yields was severely damaged under extreme cold conditions. However, studies about the transcriptional and metabolic mechanisms underlying cold stresses in wheat are limited so far.

**Results:**

In this study, 14,466 differentially expressed genes (DEGs) were obtained between wild-type and cold-sensitive mutants, of which 5278 DEGs were acquired after cold treatment. 88 differential accumulated metabolites (DAMs) were detected, including P-coumaroyl putrescine of alkaloids, D-proline betaine of mino acids and derivativ, Chlorogenic acid of the Phenolic acids. The comprehensive analysis of metabolomics and transcriptome showed that the cold resistance of wheat was closely related to 13 metabolites and 14 key enzymes in the flavonol biosynthesis pathway. The 7 enhanced energy metabolites and 8 up-regulation key enzymes were also compactly involved in the sucrose and amino acid biosynthesis pathway. Moreover, quantitative real-time PCR (qRT-PCR) revealed that twelve key genes were differentially expressed under cold, indicating that candidate genes *POD*, *Tacr7*, *UGTs,* and *GSTU6* which were related to cold resistance of wheat.

**Conclusions:**

In this study, we obtained the differentially expressed genes and differential accumulated metabolites in wheat under cold stress. Using the DEGs and DAMs, we plotted regulatory pathway maps of the flavonol biosynthesis pathway, sucrose and amino acid biosynthesis pathway related to cold resistance of wheat. It was found that candidate genes *POD*, *Tacr7*, *UGTs* and *GSTU6* are related to cold resistance of wheat. This study provided valuable molecular information and new genetic engineering clues for the further study on plant resistance to cold stress.

**Supplementary Information:**

The online version contains supplementary material available at 10.1186/s12870-022-03718-2.

## Background

Wheat (*Triticum aestivum L.*) is one of the largest planted proportion crops globally, and is essential for human nutrition and animal feed. China is one of the countries where wheat is grown most widely in the world.. Wheat is often affected by different types of typically abiotic stresses such as cold injury and drought during growth and development, directly influencing wheat’s yield [1]. Climate-driven extreme temperatures affect the nutritional and reproductive growth of wheat, which in turn leads to a further decline in yield [2].. Cold is a climatic element that astrict crop growth and affects crop yield. Plants can respond to cold stress through series of physiological and biochemical changes [3]. Understanding the response of plants to cold stress is very important for crop molecular breeding [4–7]. Low temperature can cause irreparable damage to plants. Overstimulation of electron transport systems cause the enhanced concentration of reactive oxygen species (ROS), which affects membrane lipids, causes damage to proteins and nucleic acids, and ultimately results in cell death [8, 9]. When plants have appeared too low temperatures, the contents of metabolites, such as carbon [10], sugar [11], and protein [8], will be self-redistributed as the temperature decreases. Under cold stress, the generation of osmoprotective agents (trehalose, raffinose, proline, fructose, and betaine) can stabilize the protein and cell structure, which are widely found in plant species [12, 13]. Plant responses to cold stress can also protect cell metabolism by synthesizing late embryogenesis abundance (*LEA*) protein [14, 15], which stabilize cell structure by binding to DNA or RNA to form a compact hydrogen bond structure and reduce damage caused by abiotic stress [16].

Progress has been made in developing cold-response genes to increase tolerance to abiotic stresses in crops [17–19]. The response of plants to cold stress involves multiple levels of regulation, including molecular level, transcriptional regulation, metabolic changes and response pathways. Frost-resistant stress signaling and transcriptional regulation of wheat are complex, involving at least two major pathways (frost response and vernalization) and many other processes involving large numbers of genes (calcium-binding protein (*CBP*) [20, 21], Ethylene response element-binding protein (*EREBP*) [22, 23], cold-responsive/late embryogenesis abundant (*COR/LEA*) genes [24, 25], cold- and dehydration-responsive genes (*CRT/DRE*) [26], vernalization (*VRN1, VRN2, VRN3*) [27, 28], frost resistance loci (*FR1, FR2*) [29]. In the plant genome, about 7% of the coding sequences are associated with transcription factors (TFs) [30]. Wheat genomes contain a large number of transcription factors that play essential roles in biotic and abiotic stress biological processes, such as C-repeat-binding factor (*CBF1*, *CBF2*, and *CBF3*) [27, 31, 32], C-repeat (*CRT*) [26, 33]. Cold stresses cause cells to dehydrate, so various signaling pathways preventing cell dehydration have been studied, including *MYB*, *WRKY*, APETALA 2/ethylene response element-binding protein (*AP2/EREBP*), *NAC,* and alkaline leucine (Leu) zipper (*BZIP*) families [34, 35].

New approaches are needed to uncover the molecular mechanisms of wheat cold tolerance. Omics technology offers unique opportunities to study the regulatory mechanisms of complex networks related to cold hardiness [36]. With the speediness evolution of sequencing tools, transcriptome sequencing technology has been extensively applied to clarify gene function by high-throughput analysis of genomic information, and provided a potent way to systematically study the relationship between genotype and phenotype in transcriptional regulation [37]. For example, it has been widely used in research wheat stress resistance to reveal differentially expressed genes in different biological processes, such as cold [38–40], drought [41], heat [42], and salt tolerance [43]. However, low abundance and unknown transcripts are unable to distinguish [37]. Compared with conventional chemical analysis, metabolomics was promoted using liquid chromatography/mass spectrometry (LC/MS) by detecting lots of compounds [44]. Recently, the comprehensive analysis of metabolome and transcriptome has been widely used to study the regulatory network and correlation between genes and metabolites [35, 45]. Metabonomic, a concept first proposed by Nicholson et al., is a new omics following transcriptome, genomics and proteomic-related investigations [46].

Metabolomic can be used for qualitative and quantitative analysis of the small molecular metabolites in cells and organisms at a specific physiological period, and has been certified as a mighty instrumentality for studying gene modification and environmentally induced metabolite changes [47]. Currently, metabolomic analysis has been widely used to reveal metabolites profiles changes under low-temperature stress in many plants. Kaplan et al. [48] used non-target gas chromatography-mass spectrometry (GC-MS) to study the dynamic *Arabidopsis thaliana* changes under heat and cold stress, and discovered that cold shock had a more significant effect on metabolites. Cold acclimation analysis of two genotypes (cold sensitivity and cold tolerance) in perennial plants was studied by the nuclear magnetic resonance (NMR) method, and more than 40 metabolites were identified [49]. Metabolomics analysis of tillering node in winter wheat was conducted by gas chromatography time-of-flight mass spectrometry (GC-TOF/MS) during the overwintering period, and confirmed that monosaccharides, amino acids and lipids maintained carbon and nitrogen balance [8]. Zhao et al. [50] investigate changes in gene/metabolite activity in Jing 411 by integrated transcriptomics and metabolomics analyses, and indicated that critical pathways associated with ABA/JA signaling and proline biosynthesis played important roles in regulating cold tolerance in wheat.

Nevertheless, studies about the transcriptional and metabolic mechanisms underlying cold stresses in wheat are limited. This study investigated the influence of low-temperature stress on wheat seedlings by physiological, transcriptome and metabonomics analysis of Jimai 325 and its mutant MU-134. Widely Targeted Metabolome technology was applied to reveal the metabolic changes affected by different cold stresses. The findings suggest that candidate genes and metabolites involved in the essential biological pathways might regulate frost resistance in wheat, and a metabolic regulatory network was established in response to low-temperature stress. This study deepened the understanding of the molecular mechanism of wheat adaptive response to low-temperature stress and provided valuable information for wheat frost tolerance gene breeding.

## Results

### Phenotypic variation under cold stress

**Fig. 1:**
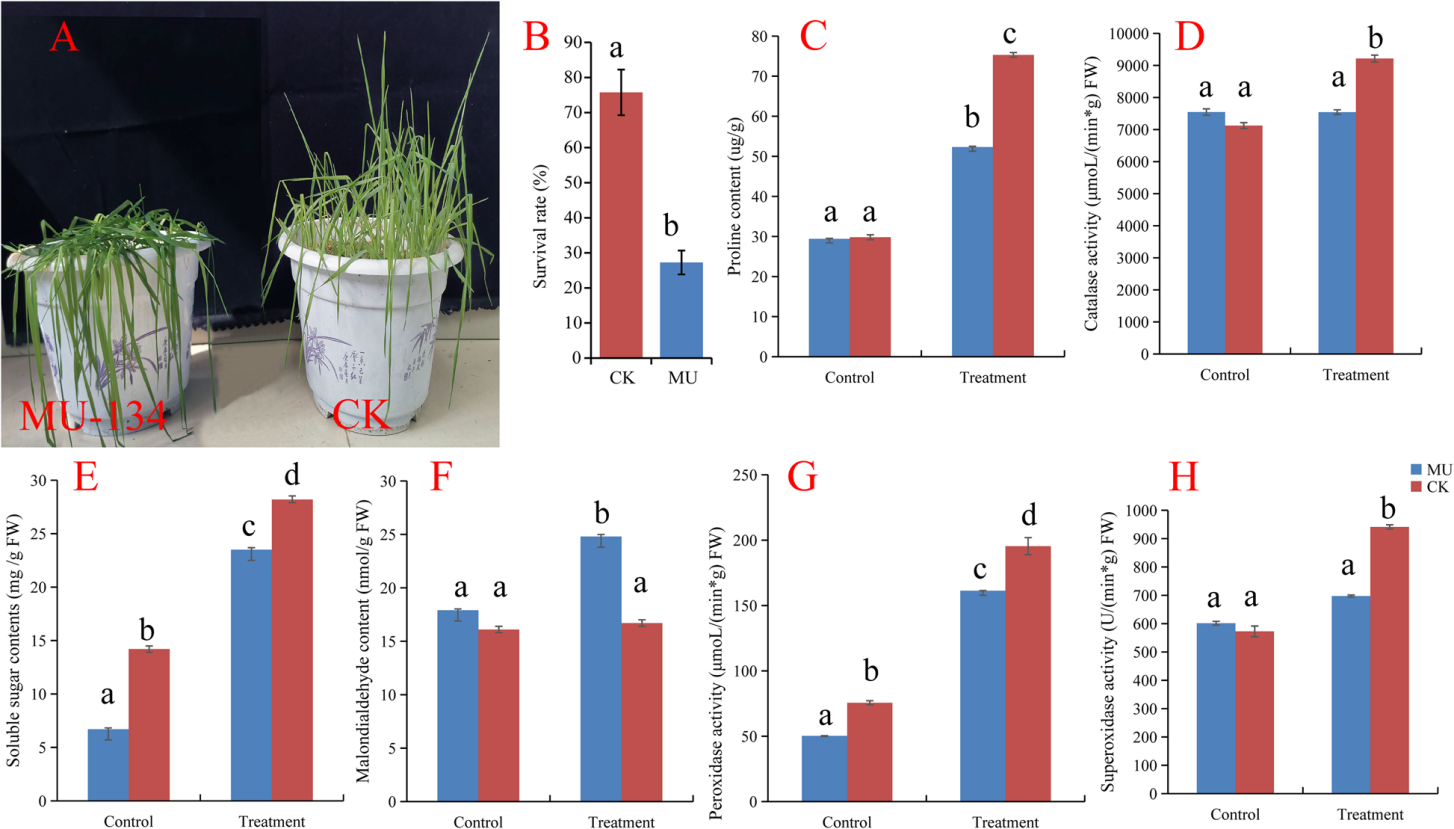
Cold Stress effects on plant development. **A** Shoot morphology. **B** Survival rate (%). **C** Proline content (ug/g). D, Catalase activity (μmoL*min*g)^−1^ FW. E, Soluble sugar contents (mg/g FW). **F** Malondialdehyde content (nmol/g FW). **G** Peroxidase activity (μmoL/(min*g) FW). **H** Superoxidase activity (U/(min*g) FW). The data are means ± SD(*n* ≥ 10). Different letters indicate significant differences between treatments and control plants from Student’s unpaired two-tailed t-test (*P* < 0.05). CK, Jimai 325; MU, MU-134

To study the responses to cold stress, 28-days-old plants were exposed to cold stresses for progressive 7-day-period treatments. The cold stress significantly affected wheat’s normal growth and development and reduced the survival rate (Fig. 1A, B). To understand the effect of low temperature stress on wheat, several physiological characteristics between the mutant plant and Jimai325 were investigated. In our research, the activity of the peroxidase (POD), proline (Pro) content and soluble sugar contents significantly increased in Jimai325 (CK) and MU-134 when treated with cold stress compared with the untreated control plants (Fig. 1C, E, G). Catalase activity (CAT) and Superoxidase activity (SOD) were significantly increased in CK when treated with cold stress compared with the untreated control plants. Still, there was no significant difference in MU-134(Fig. 1D, H). Malondialdehyde content only significantly increased in MU-134 when treated with cold stress compared with the untreated control plants (Fig. 1F).

### Global transcriptomic changes under cold stress in wheat

**Fig. 2:**
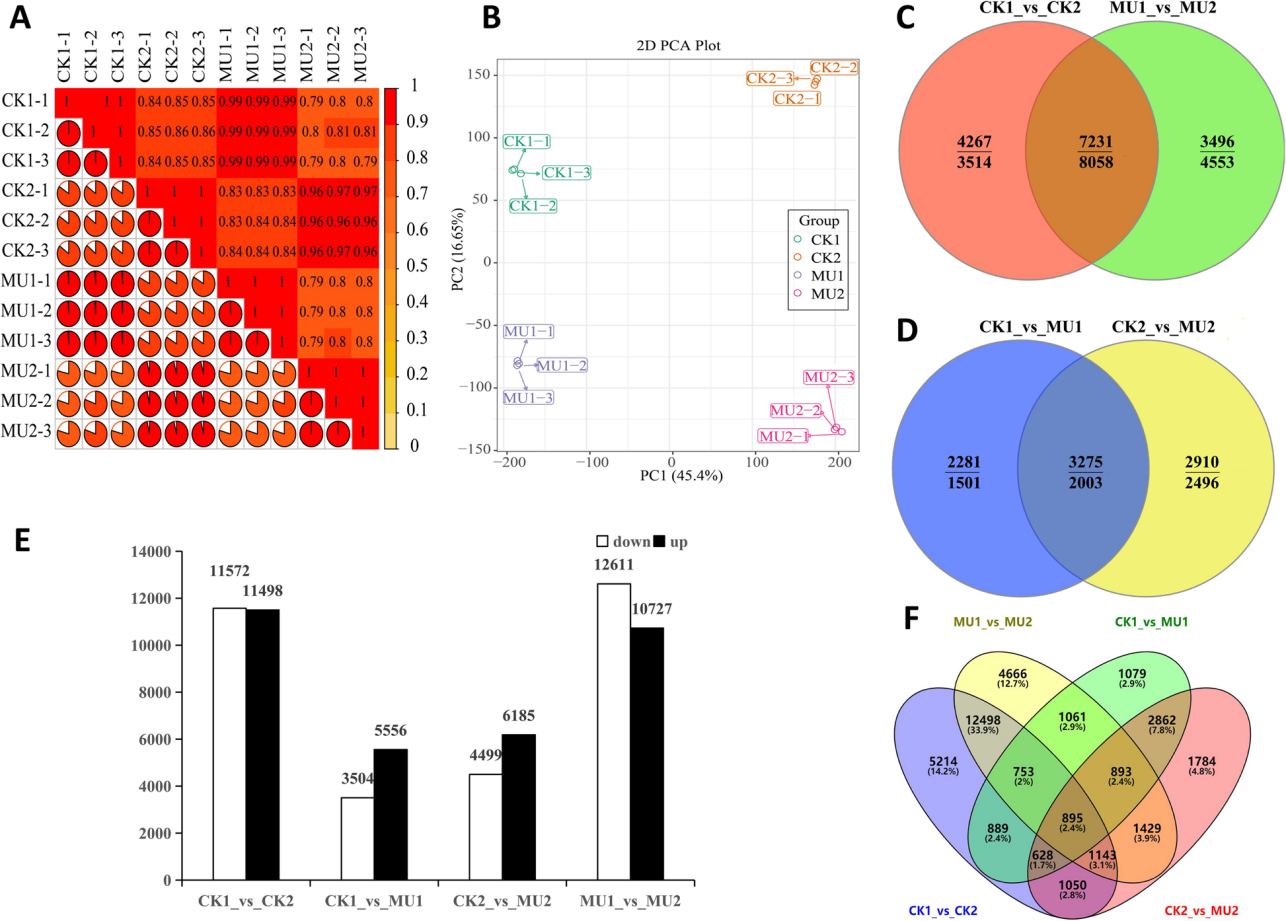
Correlation and PCA analysis of all transcripts and identification of DEGs. **A** Correlation analysis of all samples. **B** PCA plot of transcriptome results. **C** Venn diagram of DEGs before and after cold stress in the same varieties; D, Venn diagram of DEGs between varieties before and after cold stress. Above and below the horizontal line are up-regulated and down-regulated genes, respectively; **E** Numbers of DEGs between different comparisons. **F** Venn diagram of DEGs among 4 comparison groups (FDR < 0.05 and FC ≥ 2). The numbers in parentheses showed percentages concerning the total genes. CK1, before treatment Jimai 325; CK2, cold treatment Jimai 325; MU1, before treatment MU-134; MU2, cold treatment MU-134

In order to identify the cold tolerance genes, RNA-seq was performed on MU and CK samples at normal and after low-temperature treatment, respectively. A total of 162.24 Gb nucleotides containing 1,081,481,144 clean reads were generated, with an average GC content of 55.66% and Q30 > 94% (Table S1). After denovo transcriptome assembly, a total of 66,152 unigenes were obtained to differentially expressed in 12 samples, using a fold change ≥1 and stringent FDR value < 0.05 were as thresholds (Table S2). High repeatability of sequencing data from 12 samples was proved by Pair-wise Pearson’s correlation coefficients (Fig. 2A). 23,070 DEGs, 9060 DEGs, 10,684 DEGs, and 23,338 DEGs were identified at CK1_vs_CK2, CK1_vs_MU1, CK2_vs_MU2 and MU1_vs_MU2, respectively (Fig. 2E). Up-regulated genes presented at CK1_vs_MU1(5556) and CK2_vs_MU2(6185) were significantly more than down-regulated genes (3504, 4499) (Fig. 2D). In contrast, the number of down-regulated genes at CK1_vs_CK2(11,572) and MU1_vs_MU2(12,611) were much more than those up-regulated (11,498, 10,727) ((Fig. 2C, Table S3). 895 specific genes were collectively found in DEGs of CK1_vs_CK2, CK1_vs_MU1, CK2_vs_MU2 and MU1_vs_MU2 (Fig. 2F).

### GO and KEGG analysis of the DEGs

**Fig. 3:**
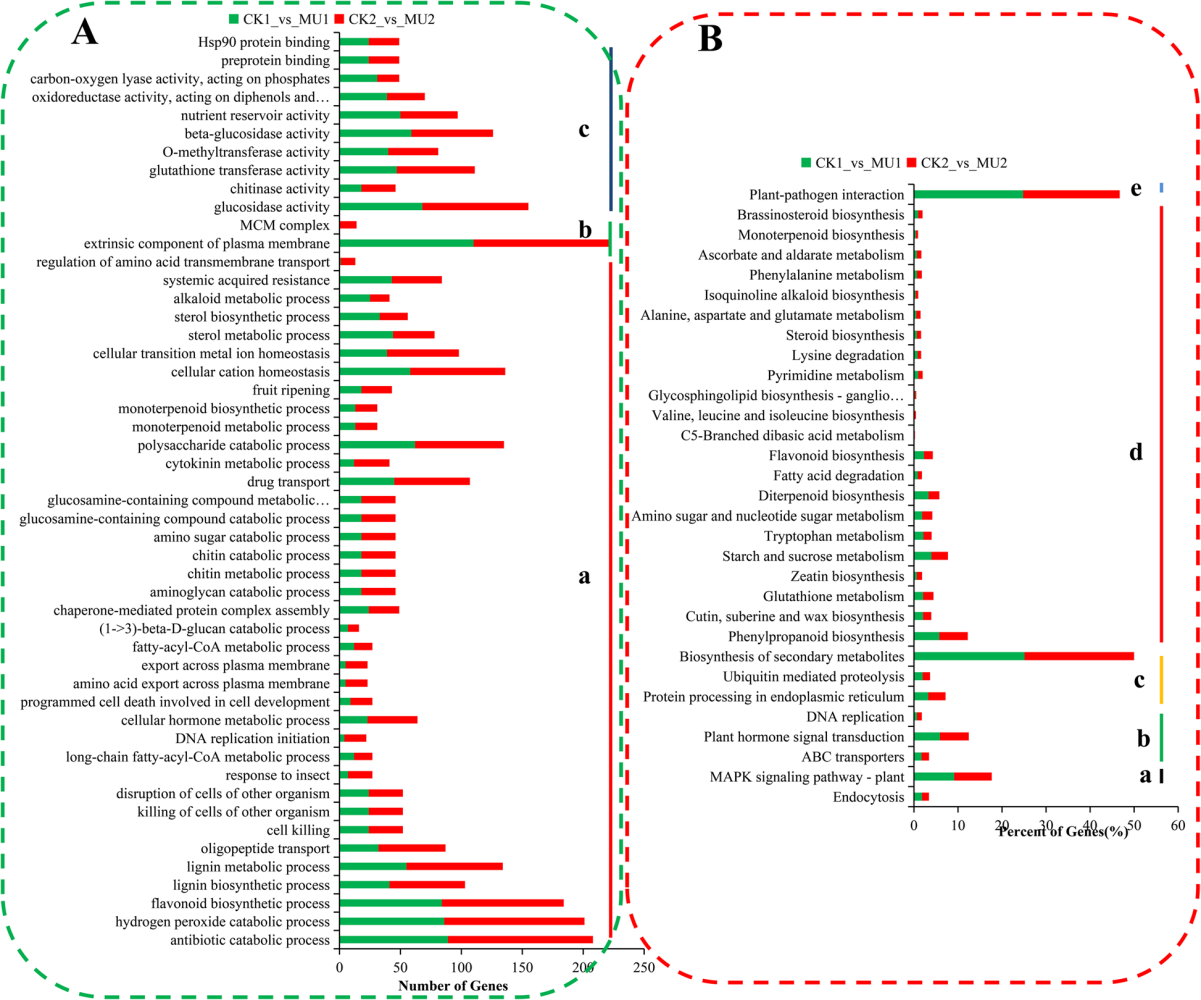
GO (A) and KEGG (B) functional classifications of the annotated unigenes in wheat. The unigenes were distributed into three GO categories: biological process (**a**), cellular component (**b**), and molecular function (**c**). The unigenes were divided into five KEGG groups: cellular processes (**a**), environmental information processing (**b**), genetic information processing (**c**), metabolism (**d**) and organismal systems (**e**)

To analyze the functions of the DEGs responding to cold between MU and CK, GO enrichment analysis was performed using the Goseq method in Blast2GO. Based on the GO annotations, 14,466 unigenes between CK1_vs_MU1, CK2_vs_MU2 were classified into 50 functional subgroups, including 38 in the biological process, 10 in molecular function and 2 in cellular component (Fig. 3A). The data showed that the major categories of biological processes are antibiotic degradation processes, hydrogen peroxide degradation processes, and flavonoid biosynthesis processes. Concerning the molecular function of the GO term, most DEGs were highly concerned with glucosidase activity, glutathione transferase activity, and beta-glucosidase activity. The Extrinsic component of plasma membrane and MCM complex were the significant categories annotated for the cellular component. The classification of GO categories was listed in Table S4.

Further, 132 and 133 pathways of the DEGs linked with the two different treatment stages between MU and CK were exposed by KEGG pathway analysis, respectively. The summary of pathways confirmed in our research was listed in Table S5. The top 31 common KEGG pathways distinguished in the MU compared with CK were grouped into five categories, including genetic information processing, cellular processes, metabolism, environmental information processing, and organismal systems (Fig. 3B). The results revealed that the biosynthesis of secondary metabolites pathway was highly enriched, followed by the plant-metabolism interaction, plant hormone signal transduction, the MAPK signaling pathway - plant, Phenylpropanoid biosynthesis and Starch and sucrose metabolism. Therefore, the DEGs annotated as “biosynthesis of secondary metabolites pathway,” “plant-metabolism interaction” and “MAPK signaling pathway - plant” were further investigated in detail.

**Fig. 4:**
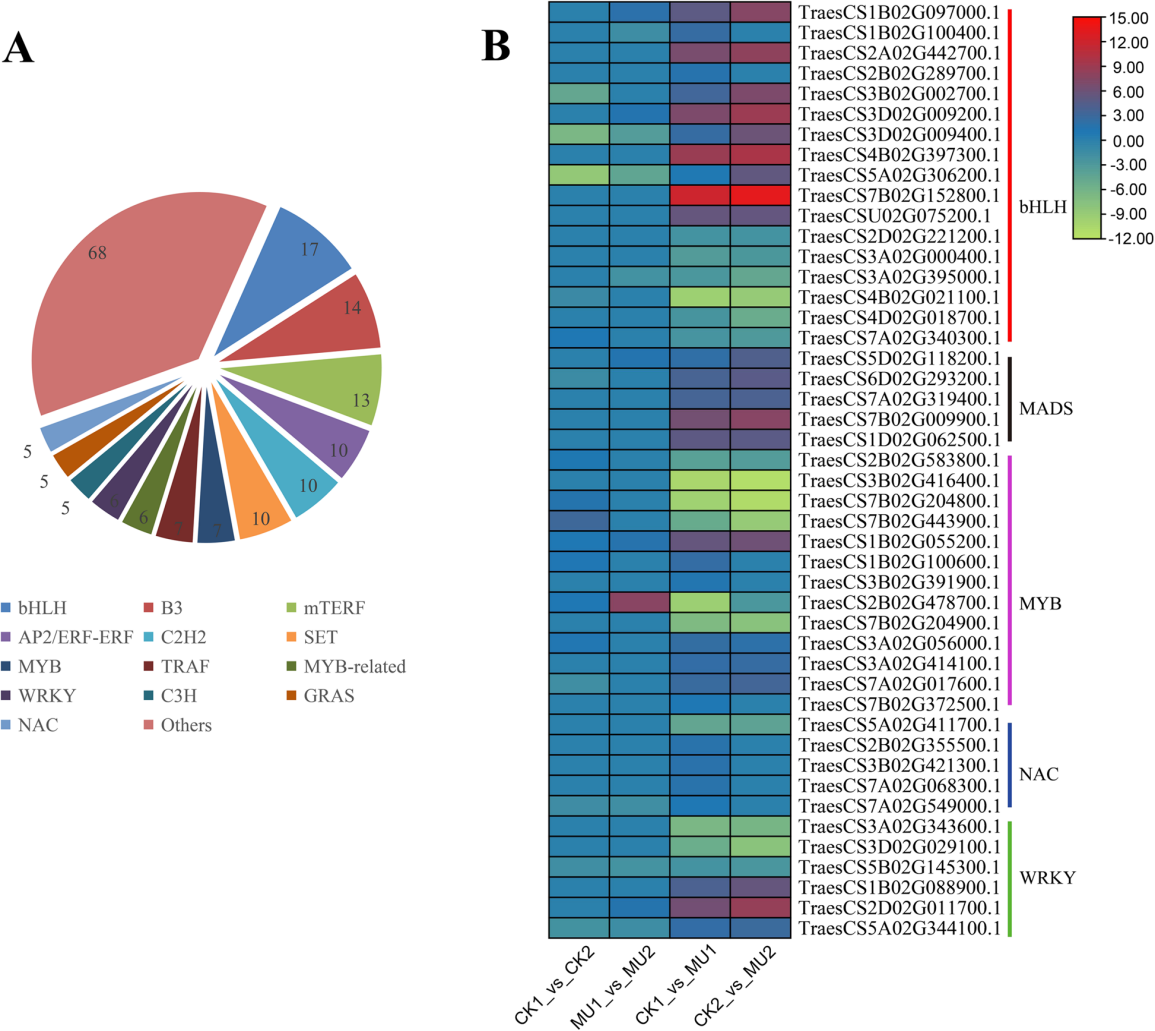
Transcription factor classification and expression profile. **A** Classification of transcription factors. **B** The expression profile of frost-resistance-related transcription factors. The numbers in the pie represent quantities. Expression was normalized; green represents low expression; red represents high expression

Transcription factors (TFs) are essential regulators in plants resistance to cold. A total of 183 significant differences TFs (FDR < 0.05 and FC ≥ 2 or FC < 0.05) were identified regulated wheat frost tolerance between MU and CK and divided into 49 categories, including 137 up TFs and 46 down TFs (Fig. 4A). ERF, B3, bHLH, C_2_H_2_, MYB, SET contain more transcription factors in different processing stages of MU and CK (Table S6). The expression levels of transcription factors related to plant frost resistance were analyzed, and which results showed the differential gene expression was significant in bHLH, MADS, MYB, and WRKY, although not always in the same direction. Concerning the general trends of TF gene expression among DEGs, bHLH were priority upregulated and WYB preferentially downregulated between MU and CK (Fig. 4B).

### Untargeted metabolite profiles of wheat responding to cold stress

**Fig. 5:**
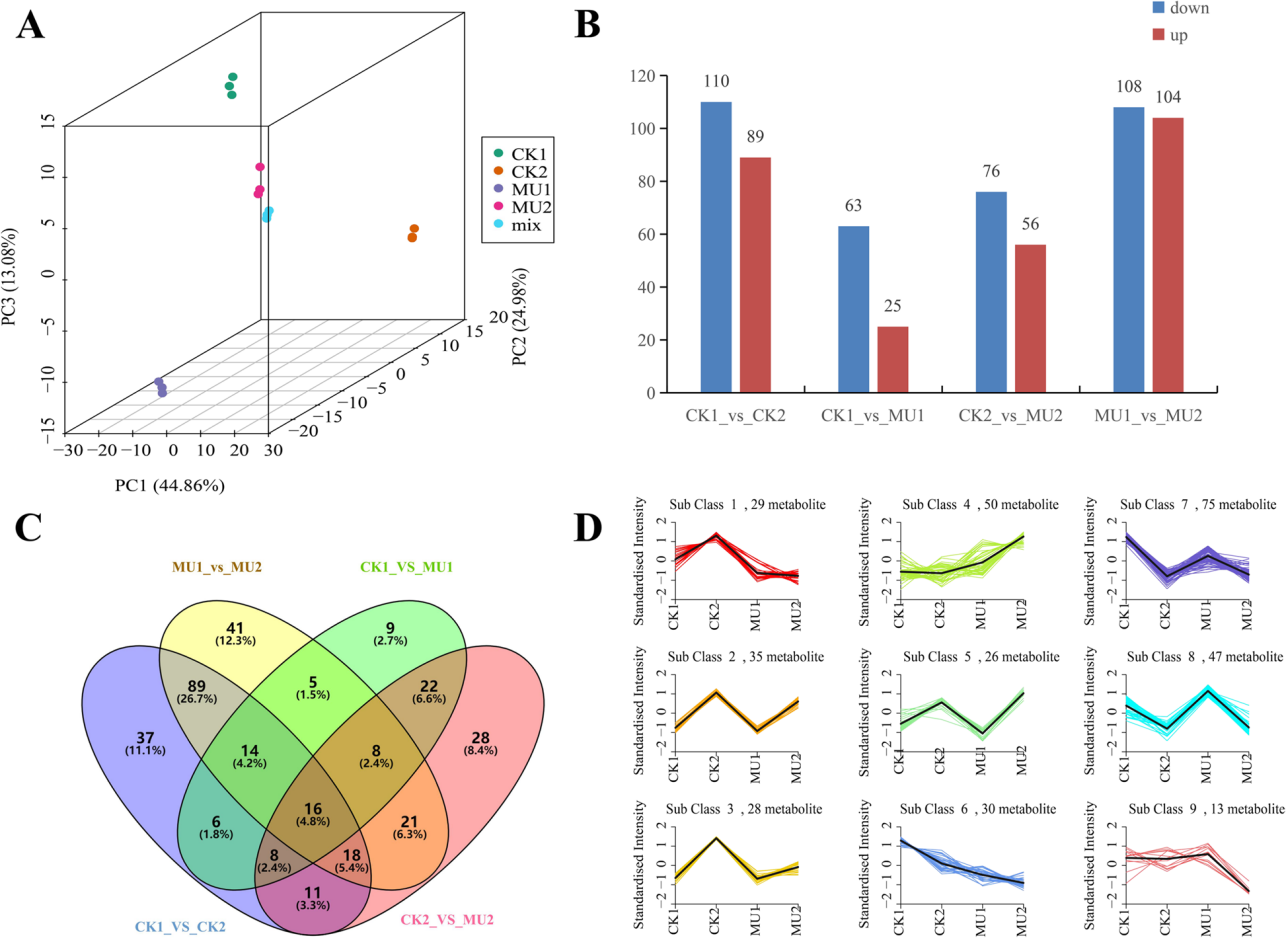
Differentially accumulated metabolites of wheat in response to cold stress. **A** PCA plot of metabolomic results. **B** Numbers of differentially accumulated metabolites under different treatments. **C** Venn diagram of differentially accumulated metabolites (FDR < 0.05 and FC ≥ 2). **D**, K-means clustering analysis of the differentially accumulated metabolites into nine clusters according to their expression profile. The cluster names and the number of metabolites for each cluster are indicated. CK1, before treatment Jimai 325; CK2, cold treatment Jimai 325; MU1, before treatment MU-134; MU2, cold treatment MU-134

To comprehensively analyze the response of wheat to low-temperature stress, we simultaneously conducted nontargeted metabolome and transcriptome analysis on the same samples. Totally 3621 peaks were obtained in UPLC-MS/MS analysis and 650 metabolites were identified using the MS2 database (Table S7). In order to determine the different metabolites of CK and MU-134 under low-temperature stress, the metabolic profiles of 12 wheat samples were analyzed by principal component analysis (PCA). The results showed obvious separation between the two samples under control and cold treatment. The principal component analysis of metabolome data was stably consistent with the transcriptomic data PCA (Fig. 5A). The variable importance for projection (VIP) value > 1.5 and FC ≥ 2 or FC < 0.05 were used to define differentially expressed metabolites. 88 metabolites were differentially accumulated compared with MU before cold treatment, with 25 up-regulated and 63 down-regulated. After cold treatment, 132 differentially accumulated metabolites were detected compared with MU, with 56 up-regulated compared with MU and 76 down-regulated, respectively (Fig. 5B). Compared with MU, 54 differentially accumulated metabolites were commonly detected in CK before low-temperature stress and after. 16 specific metabolites were collectively found in differentially accumulated metabolites of CK1_vs_CK2, CK1_vs_MU1, CK2_vs_MU2 and MU1_vs_MU2 (Fig. 5C, Table S8).

To identify metabolites with the accordance expression mode in clusters, we employed the K-means clustering algorithm, which groups the metabolites according to the similarity of the metabolome spectrum. A total of 9 clusters were confirmed (Fig. 5D), which can be divided into 5 categories: up-regulated in both CK1_vs_CK2 and MU1_vs_MU2 (class 2, 3, and 5), down-regulated in both CK1_vs_CK2 and MU1_vs_MU2 (class 6, 7, and 8), no change in CK1_vs_CK2 but upregulated in MU1_vs_MU2 (class 4), no change in CK1_vs_CK2 but down-regulated in MU1_vs_MU2 (class 9) and upregulated in CK1_vs_CK2 but no change in MU1_vs_MU2 (class 1). Among the three clusters with an overall decreasing trend of metabolite accumulation, cluster 6 metabolite was enhanced assembly in CK1 and CK2 but descended in MU1 and MU2 (Fig. 5D).

**Fig. 6:**
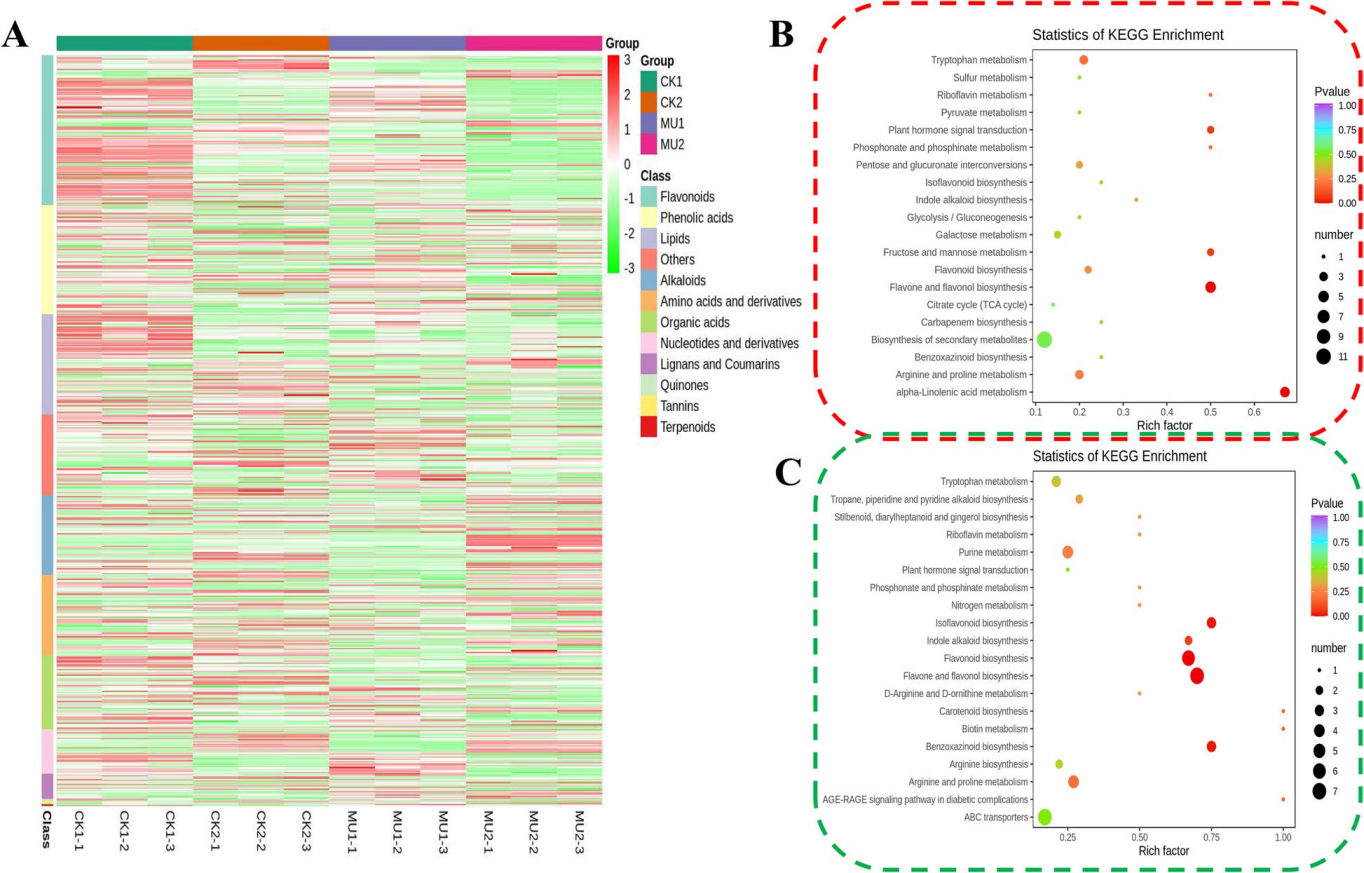
Hierarchical cluster and KEGG enrichment analysis for all metabolome samples of frost resistance traits in wheat. **A** Hierarchical cluster analysis of all samples metabolite content. Blue represents low expression and red represents high expression. **B** KEGG pathway enrichment analysis based on the differentially accumulated metabolites in MU1_vs_CK1(B) and CK2_vs_MU2 (C). The top 20 statistics of KEGG pathway enrichment were shown. The dot color represents the size of the *P*-value. The smaller the q value, the closer the color is to red. The dot size represents the number of different genes contained in each pathway

In order to identify the different metabolites between MU and CK in response to cold stress, all metabolite profiles were performed by hierarchical cluster analysis based on Pearson correlation. Within the control or cold treatment, the two samplings (MU and CK) could be detected a distinct separation. In addition, most of the differential metabolites were found to be flavonoids, termites and acids lipids (Fig. 6A). KEGG analysis of all differentially accumulated metabolites revealed the top 20 statistics of KEGG pathway enrichment. Metabolites participating in alpha-Linolenic acid metabolism, flavone and flavanol biosynthesis, Fructose and mannose metabolism, Plant hormone signal transduction and tryptophan metabolism were predominantly enriched in CK1_vs_MU1 (Fig. 6B). “flavone and flavanol biosynthesis” contained the greatest number of differentially accumulated metabolites, followed by “alpha-Linolenic acid metabolism.” Metabolites participating in flavone and flavanol biosynthesis, flavonoid biosynthesis, Benzoxazinoid biosynthesis, isoflavonoid biosynthesis and indole alkaloid biosynthesis were predominantly enriched in CK2_vs_MU2 (Fig. 6C). “flavone and flavanol biosynthesis” contained the greatest number of differentially accumulated metabolites, followed by “flavonoid biosynthesis. “.

**Fig. 7:**
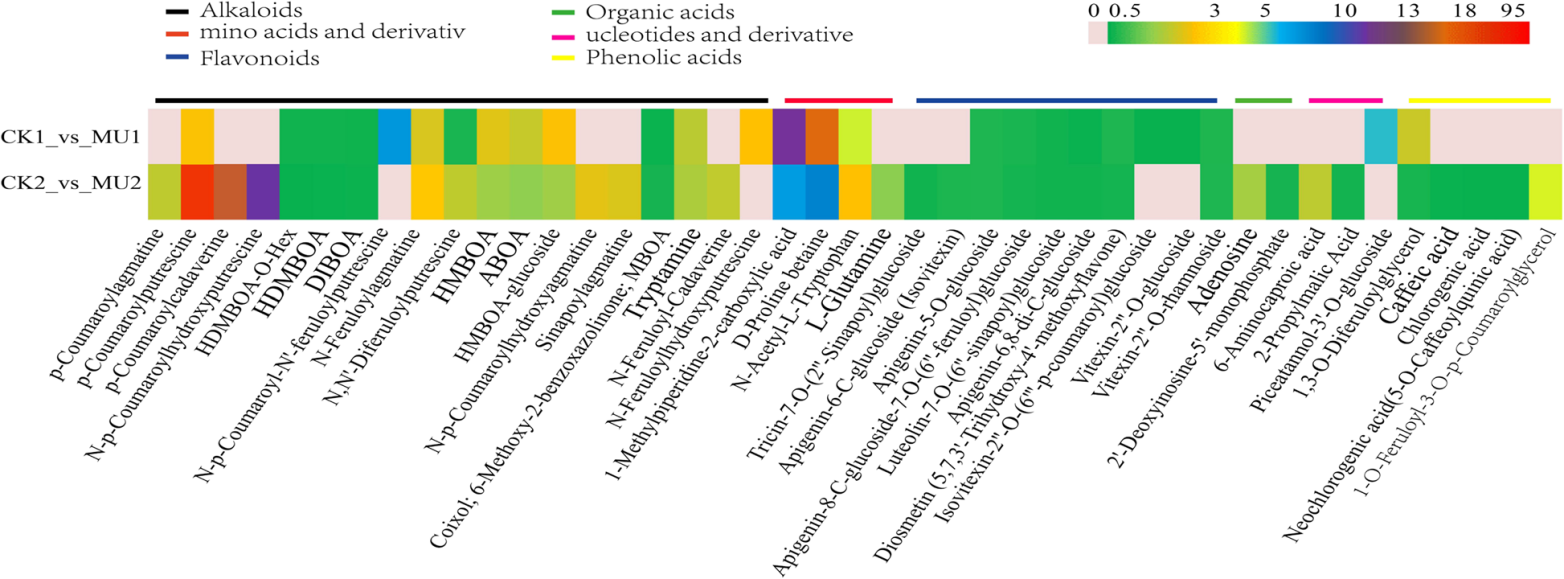
The Heatmap of key metabolites in response to the cold treatment in MU and CK. Gray represents low fold change and red represents high fold change

We also analyzed the critical metabolites of CK1_vs_MU1 and CK2_vs_MU2, and found a total of 43 metabolites with significant differences, which could be divided into six categories, including 20 up-regulated metabolites and 23 down-regulated metabolites. Class alkaloids metabolites were the most abundant, followed by class flavonoids. p-Coumaroylputrescine has the highest difference multiple as high as 95.52(Fig. 7). Table 1 displayed a list of the 10 metabolites with the highest differential accumulation. The top upregulated metabolites were primarily alkaloids, amino acids and derivativ. Compared to CK2, p-Coumaroylputrescine showed the greatest fold change in MU2, with a log^2^ fold change (FC) (MU2/CK2) value of 6.58. Chlorogenic acid, the most downregulated metabolites, had the lowest log^2^ fold change (FC) (MU2/CK2) value of − 3.01 (Table 1).

**Table 1:** List of top 10 up-regulation and down-regulation metabolites in MU vs CK

Index	Compounds	Class I	CK1_MU1			CK2_MU2		
			Fold_Change	Log2FC	Type	Fold_Change	Log2FC	Type
pmb0490	p-Coumaroyl putrescine	Alkaloids	4.43	2.15	up	95.52	6.58	up
Lmhp002772	p-Coumaroyl cadaverine	Alkaloids	–	–	–	59.29	5.89	up
pmb0508	p-Coumaroyl agmatine	Alkaloids	–	–	–	30.02	4.91	up
Lmhp002153	N-p-Coumaroylhydroxyputrescine	Alkaloids	–	–	–	17.01	4.09	up
Lmhp005550	N,N′-Diferuloylputrescine	Alkaloids	0.39	−1.34	down	3.19	1.67	up
Rfmb320	1-Methylpiperidine-2-carboxylic acid	mino acids and derivativ	51.54	5.69	up	13.82	3.79	up
Rfmb318	D-Proline betaine	mino acids and derivativ	61.77	5.95	up	14.79	3.89	up
pmp000091	1,3-O-Diferuloylglycerol	Phenolic acids	0.35	−1.50	down	3.38	1.71	up
mws0178	Chlorogenic acid	Phenolic acids				0.12	−3.01	down
mws2212	Caffeic acid	Phenolic acids				0.15	1.22	down

### Integrated analyses of transcriptomic and metabolomic changes involved in vital biological pathways

**Fig. 8:**
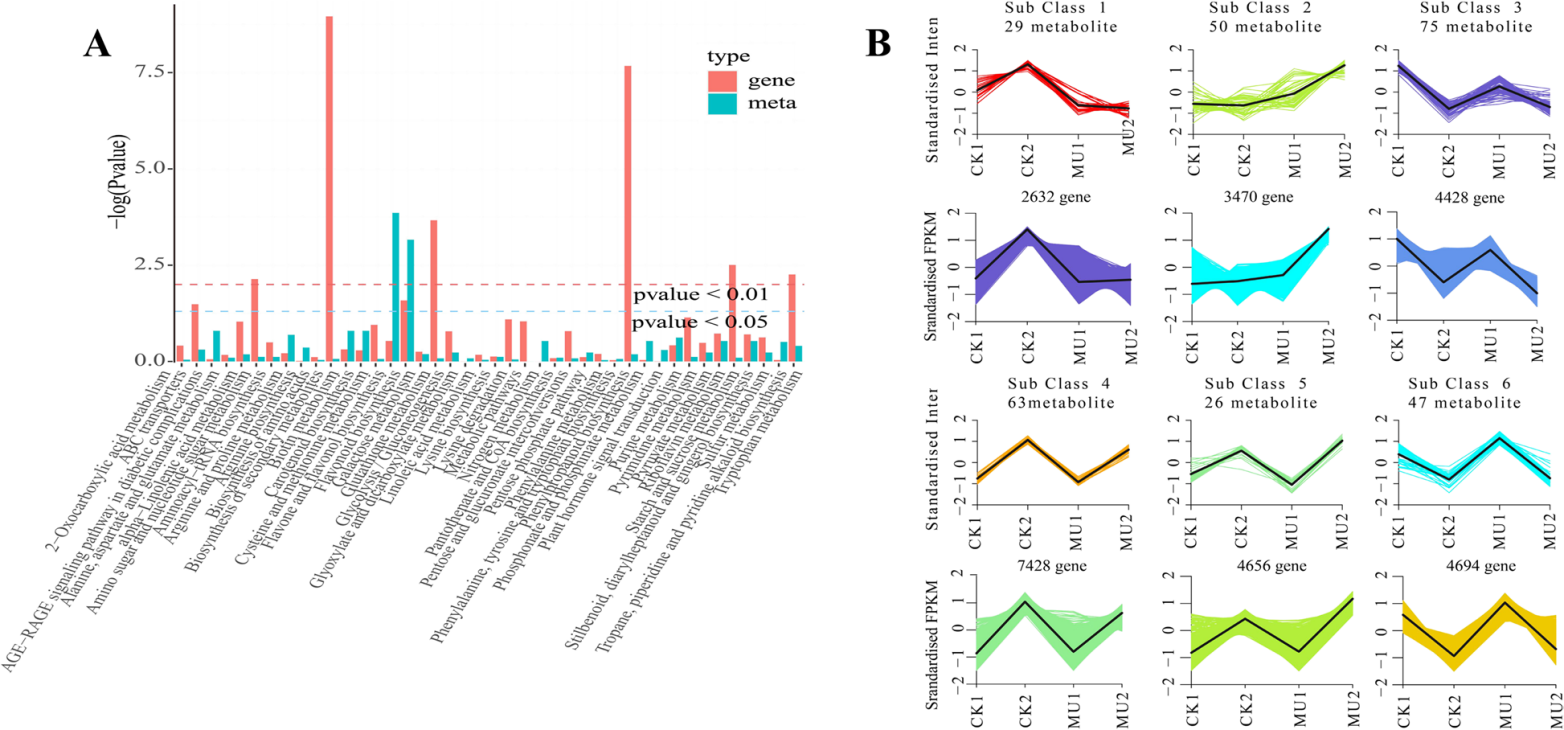
Kegg functional classifications (A) and Kmeans (B) of the differentially expressed related genes and metabolites in response to the cold treatment in MU2 and CK2

The metabolomics and transcriptome data were combined to further reveal the response of CK and MU to cold stress. We integrated analyzed all the data before and after cold stress treatment, focusing on analyzing related genes and metabolites between CK and MU after cold stress treatment. Comprehensive analysis of CK2 and MU2 showed that 12,511 genes were positively associated with 138 metabolites, including 9764 DEGs and 132 metabolites with different accumulations. The different accumulations metabolites and DEGs were enriched in the same KEGG pathways, including galactose metabolism (Fig. 8A). The DEGs were chiefly related to biotin metabolism, phenylalanine, tyrosine and tryptophan biosynthesis, glycolysis/gluconeogenesis, and starch and sucrose metabolism, suggesting that wheat may alleviate cold stress by a comprehensive series of energy metabolism and amino acid biosynthesis mechanisms. 290 metabolites and 27,308 genes in 6 clusters had consistent expression patterns by Kmeans integrated analysis of transcriptomics and metabolomics, indicating that gene expression was significantly correlated with metabolite accumulation (Fig. 8B).

**Fig. 9:**
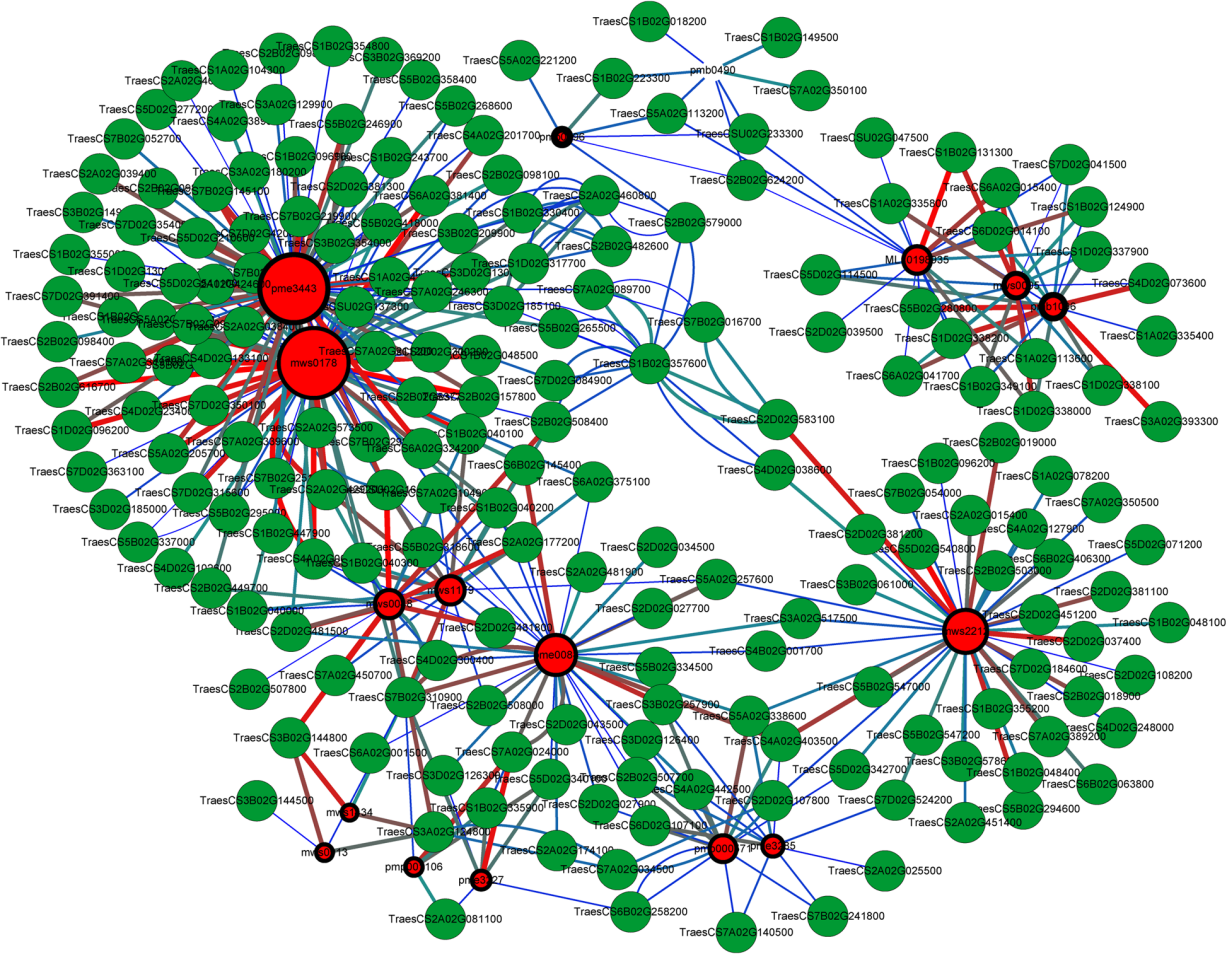
Correlation network analysis of differential metabolites and differential expression genes related to frost tolerance in wheat. The red lines indicate positive correlation; the green line indicates negative correlation, the blue line indicates the correlation is not significant. Metabolites were marked red, structural genes were green. The size of the red circle represents the number of genes associated with the metabolite. The thickness of the ring frame of metabolite group was adjusted to indicate the differential multiple of metabolites. The line thickness between nodes represents the degree of correlation between two nodes

Gene-metabolite interaction networks can help to understand functional relationships and identify novel regulatory elements. Spearman correlation method was used to calculate the correlation coefficient between DEGs and metabolites with significant differences related to the flavonol biosynthesis pathway, such as “phenylpropanoid biosynthesis,” “flavonoid biosynthesis” and “flavone and flavonol biosynthesis.” On the other hand, correlations were found for pathways related to sucrose metabolism and some amino acid biosynthesis (arginine and proline metabolism). 316 DEGs and 12 DAMs involved in flavonol biosynthesis pathway were executed Pearson correlation analysis. The result showed that 76 DEGs had strong positive correlation coefficient values (R^2^ > 0.9 and *P*-value < 0.05) with 11 metabolites, and 68 DEGs had strong negative correlation coefficient values (R^2^ < − 0.9 and P-value < 0.05) with 10 metabolites. For sucrose metabolism and some amino acid biosynthesis, 57 DEGs and 4 DAMs were carried out in Pearson correlation analysis. The result showed that 5 DEGs had strong positive correlation coefficient values (R^2^ > 0.9 and P-value < 0.05) with 3 metabolites, and 16 DEGs had strong negative correlation coefficient values (R^2^ < − 0.9 and P-value < 0.05) with 4 metabolites (Fig. 9). Further analysis revealed that the abundance of most metabolites was significantly positively correlated with the expression of four key genes, including coniferyl alcohol acyltransferase (*CFAT*), UDP-glycosyltransferase (*UDPG*), and tryptamine benzoyl transferase (*TBT*). Two genes negatively correlated with the abundance of most metabolites were flavonoid 3′ -monooxygenase (*CYP75B4*) and Eceriferum26(*CER26*).

### Flavonol biosynthesis pathway

The Kyoto Encyclopedia of Genes and Genomes (KEGG) enrichment pathways were compared and analyzed between transcriptome and metabolome of wheat for frost resistance. Multiple pathways were enriched between CK and MU, including phenylpropanoid biosynthesis, flavonoid biosynthesis, flavone and flavonol biosynthesis, starch and sucrose metabolism, and arginine and proline metabolism.

**Fig. 10:**
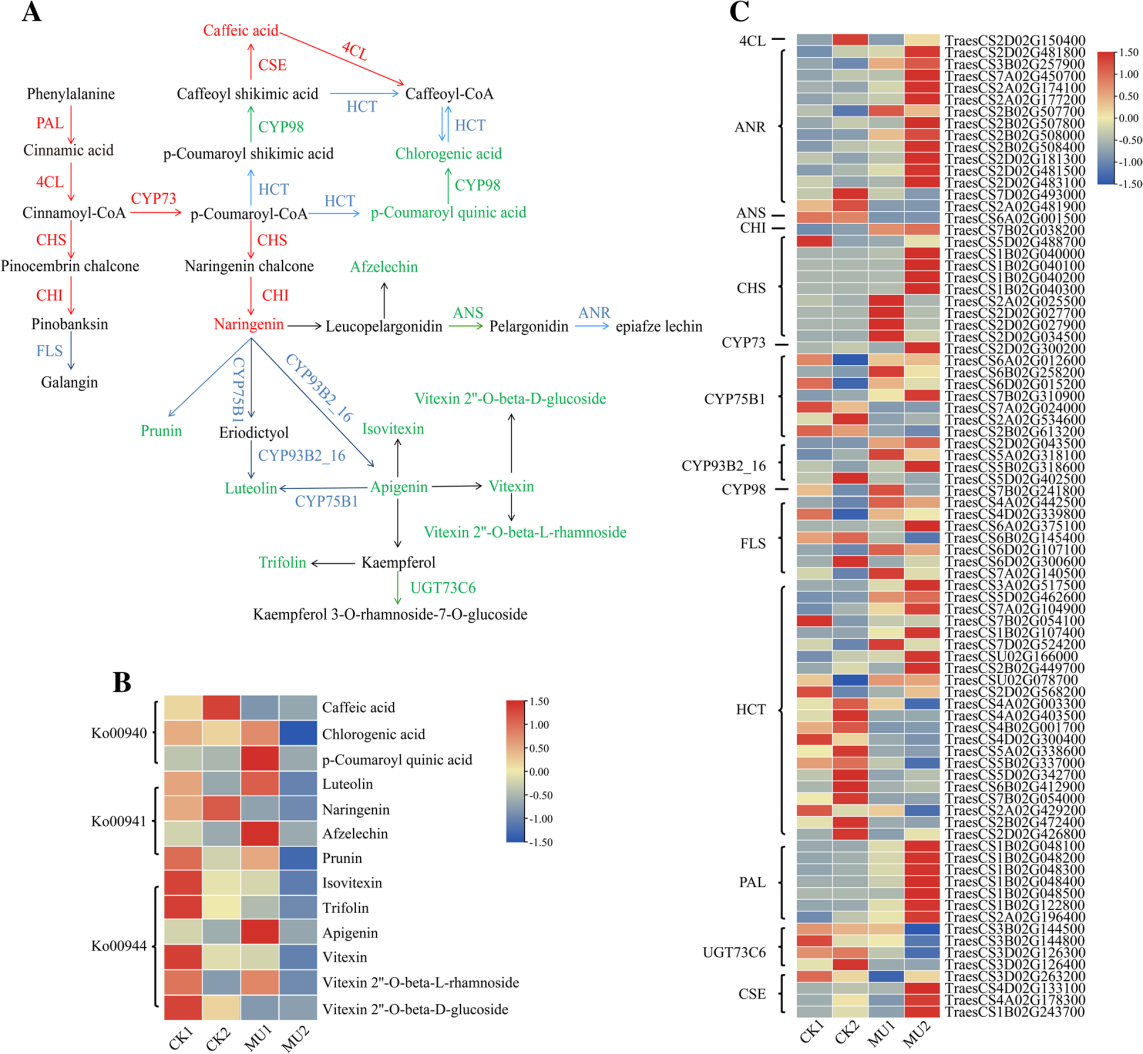
Transcript and metabolic profiling of significantly differential metabolites and DEGs in flavonol biosynthesis pathway in wheat under cold treatment. **A** The map of integrative analysis in flavonol biosynthesis metabolites and their biosynthesis-related key enzymes. Red font: significantly up-regulated differential metabolites or DEGs encoding the corresponding enzyme; green font: significantly down-regulated differential metabolites or DEGs encoding the corresponding enzyme; Blue font: significantly up/down-regulated DEGs encoding the corresponding enzyme; Black font: the enzyme encoding genes and differential metabolites detected but with no significant difference. Black arrow lines: ko00940; Blue arrow lines: ko00941; Red arrow lines: ko00944. DEGs and DAMs were investigated using the Kyoto Encyclopedia of Genes and Genomes (KEGG) to map to the possible KEGG pathway maps for the biological interpretation of systemic functions (www.kegg.jp/kegg/kegg1.html). **B** Heatmap of differential metabolites accumulation flavonol biosynthesis pathway. The color bar presented at the top right represents the level of metabolites, where red indicates the metabolites with a higher level, and blue indicates metabolites with a lower level. **C** Heatmap of DEGs enriched flavonol biosynthesis pathway. Gene expression was scaled using normalized FPKM for the mean value of three biological replicates. A color bar was presented at the top right, and the colors from blue to red indicate low to high expression. *4CL*, 4-coumarate--CoA ligase; ANS, anthocyanidin synthase; *CHI*, chalcone isomerase; *CHS*, chalcone synthase; *CYP73*, trans-cinnamate 4-monooxygenase; *CYP75B1*, flavonoid 3′-monooxygenase; *CYP93B2_16*, flavone synthase II; *CYP98*, 5-O-(4-coumaroyl)-D-quinate 3′-monooxygenase; *FG2*, Kaempferol-3-O-rutinoside; *FLS*, flavonol synthase; *HCT*, shikimate O-hydroxycinnamoyltransferase; *PAL*, phenylalanine ammonia-lyase; *UGT73C6*, flavonol-3-O-L-rhamnoside-7-O-glucosyltransferase; *CSE*, caffeoyl shikimate esterase

The flavonol biosynthesis pathway includes three pathways: “phenylpropanoid biosynthesis,” “flavonoid biosynthesis”, and “flavone and flavonol biosynthesis” [51]. In order to further elucidate the specific role and clarify the regulatory mechanism of frost resistance at the seedling stage of wheat, we have combined analysed significantly different metabolites and significantly differentially expressed genes (DEGs) in the flavonol biosynthesis pathway. The results showed that 13 significantly difference metabolites and 14 key enzymes encoded by 83 markedly DEGs were mapped onto the phenylpropanoid/flavonoids metabolic pathway (ko00940, ko00941 and ko00944), as shown in Fig. 10. The expression of 59 genes was strongly up-regulated in 83 genes, including all genes that encoded *CHI* (chalcone isomerase), *CHS* (chalcone synthase), *CYP73* (trans-cinnamate 4-monooxygenase), *4CL* (4-coumarate--CoA ligase), *PAL* (phenylalanine ammonia-lyase) and *CSE* (caffeoyl shikimate esterase) (Table S9). In the phenylpropanoid pathway (ko00940), the DEGs *PAL* (*TraesCS1B02G048100*, *TraesCS1B02G048200*, *TraesCS1B02G048300*, *TraesCS1B02G048400*, *TraesCS1B02G048500*, *TraesCS1B02G122800*, *TraesCS2A02G196400*), 4CL (*TraesCS2D02G150400*), and CSE (*TraesCS3D02G263200*, *TraesCS4D02G133100*, *TraesCS4A02G178300*, *TraesCS1B02G243700*) were up-regulated, and the corresponding metabolites of cinnamic acid and caffeic acid ignificantly increased. The up-regulation or down-regulation of 22 DEGs encoding HCT (shikimate O-hydroxycinnamoyl transferase) and *CYP98* (*TraesCS7B02G241800*) reduced the metabolites of p-Coumaroyl quinic acid and Chlorogenic acid metabolites. P-Coumaroyl coenzyme A (*CoA*) is converted to naringenin according to serial enzymes (*CHS*, *CHI*) in the “phenylpropanoid biosynthesis” pathway. Since then, afzelechin, prunin, luteolin, isovitexin, vitexin 2″-O-beta-L-rhamnoside, apigenin, vitexin, vitexin 2″-O-beta-D-glucoside, and trifolin metabolites were downregulated in the latter two pathways (ko00941, ko00944) through a series of enzymes, such as flavonoid 10′-monooxygenase (*CYP75B1*), flavone synthase II amino acid enzyme (*CYP93B2_16*), and flavonol-3-O-L-rhamnoside-7-O-glucosyltransferase (*UGT73C6*) (Fig. 10, Table S9).

### Sucrose and amino acid biosynthesis pathway

**Fig. 11:**
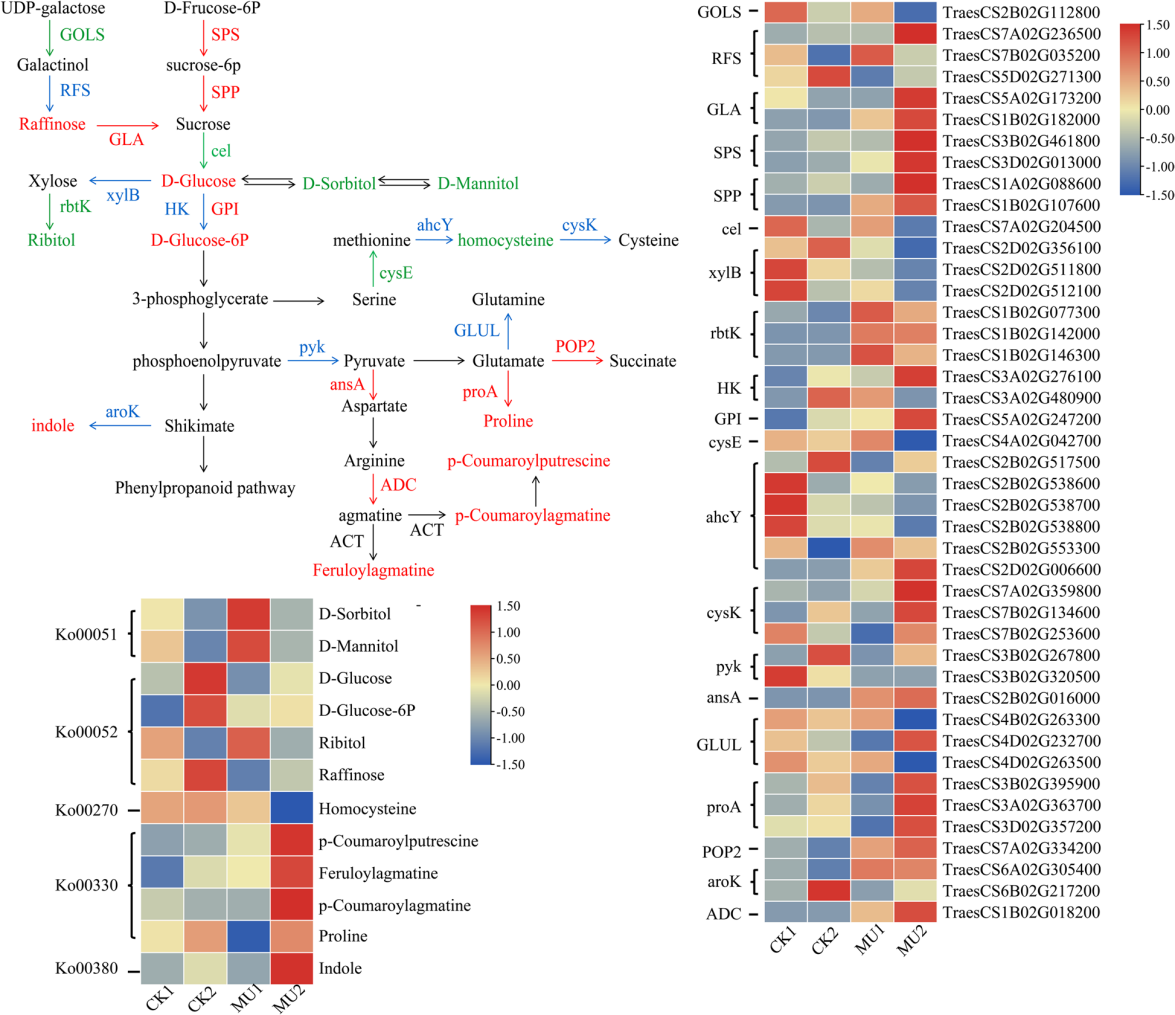
Transcript and metabolic profiling of significantly differential metabolites and DEGs through sugar and amino acids metabolic pathways in wheat under cold treatment. **A** The map of integrative analysis in sugar and amino acids metabolites and their biosynthesis-related key enzymes. Red font: significantly up-regulated differential metabolites or DEGs encoding the corresponding enzyme; green font: significantly down-regulated differential metabolites or DEGs encoding the corresponding enzyme; Blue font: significantly up/down-regulated DEGs encoding the corresponding enzyme; Black font: the enzyme encoding genes and differential metabolites detected but with no significant difference. DEGs and DAMs were investigated using the Kyoto Encyclopedia of Genes and Genomes (KEGG) to map to the possible KEGG pathway maps for the biological interpretation of systemic functions (www.kegg.jp/kegg/kegg1.html). **B** Heatmap of differential metabolites accumulation in sugar and amino acids biosynthesis pathways. The color bar presented at the top right represents the level of metabolites, where red indicates the metabolites with a higher level, and blue indicates metabolites with a lower level. **C** Heatmap of DEGs enriched in sugar and amino acids biosynthesis pathways. Gene expression was scaled using normalized FPKM for the mean value of three biological replicates. A color bar was presented at the top right, and the colors from blue to red indicate low to high expression. *SPP,* sucrose-6-phosphatase; *ADC*, arginine decarboxylase; *rbtK*, D-ribulokinase; *ansA*, L-asparaginase; *GOLS*, inositol 3-alpha-galactosyltransferase; *ahcY*, adenosylhomocysteinase; *xylB*, xylulokinase; *HK*, hexokinase; *pyk*, pyruvate kinase; *proA*, glutamate 5-kinase; *SPS*, sucrose-phosphate synthase; *cysE*, serine O-acetyltransferase; *GLUL*, glutamine synthetase; *GLA*, alpha-galactosidase; *GPI*, glucose-6-phosphate isomerase; *aroK*, shikimate kinase; *cel*, endoglucanase; *RFS*, raffinose synthase; *POP2*, 4-aminobutyrate---pyruvate transaminase; *cysK*, cysteine synthase

To better analyze the cooperation between differentially expressed genes (DEGs) and metabolites, we also chose some DEGs and metabolic associated with sugar and amino acid pathways to draw the sketch map directing the difference between CK and MU after cold treatment (Fig. 11). The results showed that 12 significantly different metabolites and 19 key enzymes encoded by 43 markedly DEGs were mapped onto the sucrose and amino acid biosynthesis pathway (ko00051, ko00052, ko00270, ko00330 and ko00380), as shown in Fig. 11. In the 43 DEGs, the expression of 21 genes was strongly up-regulated, including all genes that encoded alpha-galactosidase (*GLA*), sucrose-phosphate synthase (*SPS*), sucrose-6-phosphatase (*SPP*), glucose-6-phosphate isomerase (*GPI*), L-asparaginase (*ansA*), glutamate 5-kinase/glutamate-5-semialdehyde dehydrogenase (*proA*), 4-aminobutyrate-pyruvate transaminase (*POP2*), arginine decarboxylase (*ADC*). The expression of 20 genes was strongly down-regulated, including all genes that encoded inositol 3-alpha-galactosyltransferase (*GOLS*), endoglucanase (*cel*), xylulokinase (*xylB*), D-ribulokinase (*rbtK*), pyruvate kinase (*pyk*) and cysteine synthase (*cysE*) (Table S10).

In the galactose metabolism pathway (ko00052), the DEGs *RFS* (raffinose synthase, *TraesCS7A02G236500*, *TraesCS5D02G271300*) were up-regulated, and the level of corresponding metabolites of raffinose markedly increased. Meanwhile, the DEGs of *SPS* (*TraesCS3B02G461800*, *TraesCS3D02G013000*), *SPP* (*TraesCS1A02G088600*, *TraesCS1B02G107600*), *GLA* (*TraesCS5A02G173200*, *TraesCS1B02G182000*) and *GPI* (*TraesCS5A02G247200*) were significantly up-regulated resulted in a increase in D-glucose and D-glucose-6p metabolites. Whereas, the ribitol metabolite was observed significantly reduce in the CK vs. MU comparison due to *rbtk* (*TraesCS1B02G077300*, *TraesCS1B02G142000* and *TraesCS1B02G146300*) down-regulated. In the cysteine and methionine metabolism pathway (ko00270), the down-regulated of DEGs *cysE* (*TraesCS4A02G042700*) and *ahcY* (adenosylhomocysteinase, *TraesCS2B02G517500*, *TraesCS2B02G538600*, *TraesCS2B02G538700*, *TraesCS2B02G538800*) caused the homocysteine significantly decline. Strikingly, the homocysteine level decreased more than 7685-fold in the CK2 vs. MU2 comparison, whereas only a 3-fold decrease was observed in the CK1 vs. MU1 comparison. In the arginine and proline metabolism pathway (ko00330), the up-regulation of DEGs encoding *proA* (*TraesCS3B02G395900*, *TraesCS3A02G363700* and *TraesCS3D02G357200*) and *POP2* (*TraesCS7A02G334200*) resulted in a increase in proline metabolites. Since then, p-Coumaroylputrescine, Feruloylagmatine and p-Coumaroylagmatine metabolites were up-regulated through a series of enzymes, such as *ansA* (*TraesCS2B02G016000*) and *ADC* (*TraesCS1B02G018200*). Significantly, the level of p-Coumaroylagmatine decreased more than 30-fold in the CK2 vs. MU2 comparison (Fig. 11, Table S10).

### Validation of differentially expressed proteins at the transcription level

**Fig. 12:**
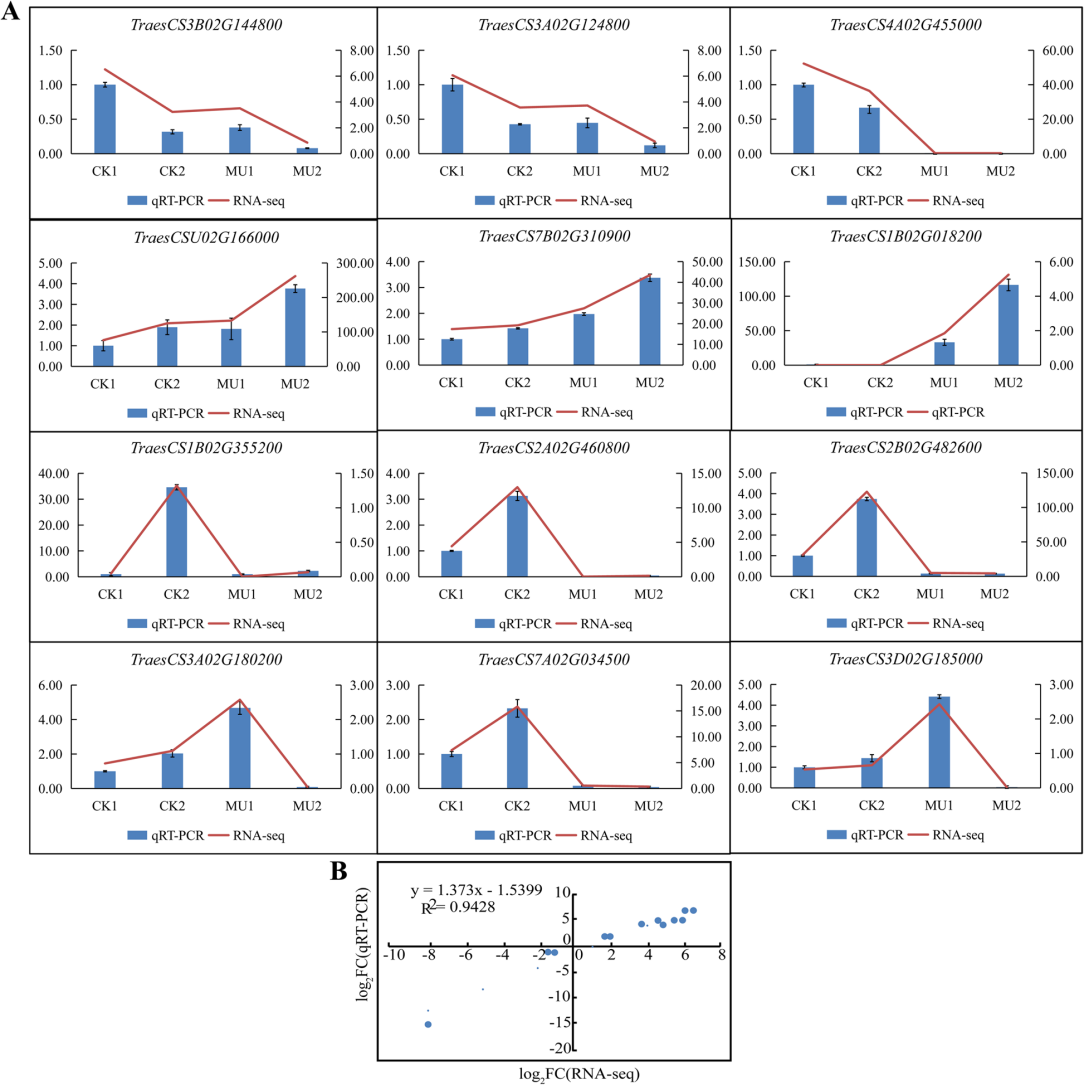
The relative expression levels of twelve selected DEGs were compared by RNA-seq and qRT-PCR. **A** Expression patterns of the 12 genes involved in the Cold stress of wheat. **B** correlation analysis based on RNA-Seq and qRT-PCR data. The line chart shows the gene expression level from the transcriptome (FPKM); The qRT-PCR expression levels were calculated as a ratio relative to the level of expression of CK1, which was set as 1. Bars indicate means ± standard deviations (SDs) of at least three independent biological replicates

To verify the reliability of expression levels in RNA-seq data, quantitative real-time PCR (qRT-PCR) was applied to validate the twelve differentially expressed frost-resistance related genes (Fig. 12A). Correlation analysis further exhibited a significant correlation (correlation coefficient of 0.94) between RNA-Seq and qRT-PCR (Fig. 12B). The qRT-PCR results corroborated that the expression trends of twelve DEGs were highly consistent with the transcriptome data and supported the credibility of RNA-seq data. *UGT73C6* (*TraesCS3B02G144800*), *UGTs* (*TraesCS3A02G124800*) and *GSTU6* (*TraesCS4A02G455000*) showed downregulation trend after receiving cold treatment, and the expression level of MU was significantly lower than CK. However, *HCT* (*TraesCSU02G166000*), *CYP75B1* (*TraesCS7B02G310900*) and *ADC* (*TraesCS1B02G018200*) were verified up-regulated after cold treatment, and the expression level of MU was significantly higher than that of CK. After cold treatment, *POD2* (*TraesCS1B02G355200*), *Tacr7* (*TraesCS2A02G460800*, *TraesCS2B02G482600*) and *GSTU6* (*TraesCS7A02G034500*) showed a sharp up-regulation trend of CK, but the expression levels of MU were very low before and after cold treatment. The relative expression of *POD72* (*TraesCS3D02G185000*, *TraesCS3A02G180200*) in MU1 was found significantly higher than that in CK1 and CK2. After cold treatment, the expression of these two DEGs was up-regulated in CK and decreased extremely to close to zero in MU. We believe that candidate genes *POD*, *Tacr7*, *UGTs* and *GSTU6* were related to frost resistance of wheat and could be utilized as transgenic or molecular marker-assisted selection to promote frost resistance of wheat. We will proceed genetic transformation of candidate genes in wheat to verify further.

## Discussion

Large numbers of DEGs were found in leaves between the wild-type and mutants before and after cold treatment. GO enrichment analysis shows that these DEGs were related to large-scale biological processes and molecular functions, such as hydrogen peroxide catabolic, extrinsic component of plasma membrane, and flavonoid biosynthetic process, which were expected, but also related to other pathways due to interaction between elements. Compared with MU1, the number of DEGs in CK1 was 9060 (5556 up-regulated and 3504 down-regulated). In contrast, cold treatment had the greatest effect on wheat transcriptome, with 10,684 DEGs changed (6185 up-regulated and 4499 down-regulated; Fig. 3E). Many genes were found in catabolic, extrinsic component of plasma membrane and flavonoid biosynthetic process before and after cold treatment. These results indicated that the frost resistance of wild-type and mutant wheat in this experiment was extremely related to plasma membrane, oxidative decomposition and flavonoids, which was consistent with previous research results [52–55], but also involved in sugar and energy metabolism [56]. In addition, these DEGs were mapped to KEGG pathways. The four largest KEGG categories were “Biosynthesis of Secondary metabolites,” “MAPK signaling Pathway-Plant,” “plant hormone signal” and “Phenylpropanoid biosynthesis” (Fig. 3B). These results were also consistent with previous studies on biological processes [57, 58]. However, there were also many genes enriched in the “plant-interaction” pathway, because low temperature significantly changed the membrane permeability of plants leading to a decrease in plant resistance to pathogens [59].

Transcription factors (TFs) also play an important role in low-temperature stress response of plants, such as *DREB* [60], *WARY* [61], *NAC* [62], *ICE* [63] and *MAD* [64]. Several transcription factors induced by low temperature stress were also identified, such as *TraesCS2D02G011700* (*WRKY*), *TraesCS3B02G416400* (*MYB*) and *TraesCS7B02G152800* (*bHLH*). These transcription factors induced by low-temperature stress may play a critical role in the frost resistance mechanism of wheat. Several key transcription factors directly regulate frost resistance of wheat in different ways. However, the number of transcription factors regulating plant frost tolerance in specific plants is minimal, and the relationship between transcription factors has not been thoroughly studied. In this study, transcriptome sequencing data provided the basis for distinguishing regulatory networks and novel regulatory factors involved in frost resistance of wheat (Fig. 4). As the second-largest transcription factor in plants, *bHLH* transcription factor plays an essential role in plant growth and development, physiological metabolism and stress response. It mainly regulates the expression of related genes through the interaction between specific amino acids and target genes. *NtbHLH123* transcription factor in tobacco enhances tolerance to cold stress through regulatory pathways and reactive oxygen homeostasis [65]. *MYB* and *bHLH* families are believed to regulate plant secondary metabolism and stress responses according to combinatorial interactions of *MYB* and *bHLH* proteins or multi-protein complexes formed of different subgroups of *bHLH* proteins [66, 67]. In this study, *TraesCS7B02G152800* (*bHLH*) and *TraesCS3B02G416400* (*MYB*) were highly induced by cold stress. This discovery is a living example of cooperation between different transcription factors to regulate plant stress tolerance. The expression of *TraesCS7B02G152800* was significantly up-regulated under cold stress, and the expression of *TraesCS3B02G416400* was significantly down-regulated under cold stress, which indicated that the two genes were related to the difference of frost tolerance between two wheat varieties.

Metabolomics can define and quantify the small molecular weight metabolites in biological cells, and has been widely used to study the accumulation patterns of metabolites. In addition, the changes of genes and metabolites in different tissues were revealed by using this technique. Metabolites are intermediate and final products that regulate plant growth and development. The total number of metabolites in plants is as high as 200,000, making them an ideal target for studying biosynthesis regulation [68]. In recent years, the genetic mechanism of plant metabolite changes has been studied [69, 70]. We detected 650 metabolites that could be annotated in MS2 database using the extensive targeted metabolomics method, and the number of detected metabolites was higher than that of previous studies on frost resistance of wheat [71]. The four largest KEGG categories associated with the expression of differential metabolites were “flavone and flavonol biosynthesis,” “flavonoid biosynthesis,” “isoflavonate biosynthesis” and “plant hormone signal transduction.” These results are consistent with previous studies on plant frost resistance biological processes [57]. Ten metabolites were detected with significant difference in frost resistance at seedling stage, of which 8 were increased and 2 were decreased. They fall into three main categories:alkaloids, mino acids and derivativ, and phenolic acids. Notably, the levels of two alkaloids, p-coumaroyl putrescine and p-coumaroyl cadaverine, were significantly increased by 95 and 59 fold, respectively. Yu et al. [72] found that p-coumaroyl glucoside is one of the primary differential accumulative metabolites of maize frost tolerance. Therefore, we believe that the accumulation of p-coumaroyl may be a metabolite formed during plant resistance to low-temperature stress.

Comparative analysis of metabolomics and transcriptome can facilitate interpreting biological processes and mechanisms involved in regulatory responses. In the process of low-temperature stress, plants will mobilize a significant number of factors for defense, especially metabolites. These reactions first require ample energy supply. Therefore, when low-temperature stress occurs, the energy metabolism of plants increases significantly [73]. Most energy of plants comes from respiration, and the substrate of respiration is glucose. The regulation of enzymes involved in sucrose synthesis and decomposition is more complex at low temperatures. Sucrose phosphate synthase (*SPS*) and sucrose-6-phosphatase (*SPP*) catalyze sucrose synthesis and their activity reflects the ability of sucrose biosynthesis pathway. The research by Guy et al. [74] showed low-temperature stress can improve the activity of *SPS* in spinach leaves. In this study, both *SPS* and *SPP* were up-regulated, which may be caused by cold stress in order to compensate for the effects of low temperature. In our study, both wild-type and mutant glucose content was higher after cold treatment than before. Interestingly, the glucose content of the wild-type was 2.4 times higher than that of the frost-sensitive mutant, and the corresponding phosphate isomerase (*GPI*) gene was also up-regulated 4.1 times. One of the reasons may be that plants regulate osmotic potential and enhance cell water retention through carbohydrate accumulation. Second, other protective substances and energy are generated through glucose metabolism. Thus, lines with poor frost resistance may exhibit lower sugar accumulation. Raffinose family (*RFOs*), as the second group of sugars in plants, plays an essential role in plant growth and development and abiotic stress resistance. Studies show that sugar beet produce an oligosaccharide raffinose to protect tissue from freezing damage in low-temperature environment [75]. Overexpression of *AnGolS1* in tomato can increase the contents of inositol galactoside, sucrose and glucose, and improve frost resistance of tomato [76]. In this study, up-regulated expression of raffinose synthase (*RFS*) resulted in increased accumulation of raffinose metabolites.

Amino acids are essential for protein synthesis, and previous studies have shown that amino acid metabolism plays an integral role in enhancing plant abiotic stress tolerance. Proline is an osmotic regulator involved in plant response to abiotic stress. It can also induce the expression of cold stress response and enhance the cold tolerance of wheat [71]. In our study, under cold treatment, the expression levels of *ProA* and *POP2* genes were up-regulated, leading to significantly higher proline content of CK and MU than before treatment, which was also consistent with the results of physiological indicators determined by us. *AnsA* and *ADC* significantly up-regulated p-Coumaroyl putrescine, Feruloyl agmatine, and p-Coumaroyl agmatine metabolite accumulation. The increase of proline, P-Coumaroyl putrescine, Feruloyl agmatine, and P-Coumaroyl agmatine reduced cold stress damage on wheat. These results strongly suggest that the accumulation of osmoprotectants (soluble carbohydrates, proline and amino acids) contributes to the improvement of cold resistance of plants [77].

Environmental stress can induce biosynthesis of secondary metabolites, such as phenylpropanoid, flavonoids, and flavone and flavonol biosynthesis [78]. The phenylpropanoid biosynthetic pathway is one of the most critical metabolite pathways in plants, producing a large number of secondary metabolites, such as lignin and flavonoids [79]. Lignification of plant tissues is a stress perception and signal transduction mechanism that can prevent freezing injury and cell collapse [78]. Recent studies have shown that the expression of structural genes， such as Chalcone synthase (*CHS*) and chalcone isomerase (*CHI*)，were induced by cold stress in phenylpropanoid pathway. Therefore, the accumulation of flavonoids and lignin can adapt plants to low-temperature environment [80]. This study found that some frost-response genes involved in the phenylpropanoid metabolism biosynthesis were expressed higher after low-temperature treatment than before. For example, PAL is a rate-limiting enzyme that catalyzes the deamination of phenylalanine to cinnamic acid and is the entry point for synthesizing all phenylalanine. Compared with before the treatment, the transcription level of 7 PALs was higher after treatment, which allowed more carbon flux to enter the phenylpropanoid pathway, leading to increased accumulation and better frost tolerance. As a branch product of phenylpropanoid metabolism, flavonoids are essential and affect basic metabolism and stress resistance in plants. When plants suffer adversity, the excessive accumulation of oxygen free radicals caused by metabolic disorders in the body is removed by a large number of flavonoids synthesized by stress, and the antioxidant activity of plants is enhanced [81]. It has been confirmed that flavonoid accumulation in *Arabidopsis* and *Spinach* was positively correlated with plant stress resistance [82, 83]. The expression levels of genes related to flavonoid synthesis were significantly up-regulated in the samples treated with low temperature, confirming that flavonoid synthesis enhanced the low-temperature tolerance of wheat leaves and reduced the damage caused by low temperature. However, flavonoid metabolite content was no change in this study, and only the decrease of metabolites of Naringenin, Afzelechin and Prunin in the flavonoid pathway was detected. In the present study, we also found that Vitexin 2″-O-beta-L-rhamnoside, Vitexin,Vitexin 2″-O-beta-D-glucoside after cold treatment metabolite accumulation were decreased. Kirakosyan [84] found that the metabolites content of Vitexin 2 “-O-rhamnoside in hawthorn increased 1.68 times after cold treatment, which may be caused by different crops and materials.

The frost resistance related genes found in this study provide reference for regulating frost resistance of wheat and have potential agricultural application value. However, the functions of most of the DEGs or DAMs found in this study remain largely unknown, and we will continue to study the functions of these genes and metabolites.

## Conclusions

In summary, the differences between frost-resistant material CK and frost-sensitive mutant MU were analyzed by physiological, metabolomics and transcriptomics. The underlying mechanisms between gene expression and metabolite biosynthesis were discussed and suggested that the biosynthesis of flavonol, sucrose and amino acid may play an essential role in wheat resistance to cold. This research provides valuable molecular information for frost resistance mechanisms in wheat, and will significantly promote the selection of frost-resistant varieties by biomarker-assisted selection. The critical regulatory genes of flavonol, sucrose and amino acid biosynthesis and their roles in frost resistance need to further explore and verify.

## Materials and methods

### Plant growth conditions and stress treatments

The MU-134 mutant utilized in this study was isolated from EMS (ethyl methanesulfonate) mutated population (3219 lines) originated by Dr. Liangjie Lv from a high yield, drought-resistant cultivar Jimai 325 cultivated by the Institute of Cereal and Oil Crops, Hebei Academy of Agriculture and Forestry Sciences (HAAFS). All plants were planted within the experimental plot at HAAFS in Shijiazhuang, China. Jimai 325 (CK) and MU-134 (M7 generation) individuals were planted in parallel and collected.

Seeds, Jimai 325 and mutant MU-134, were sterilized in 10% (v/v) H_2_O_2_ for 25 min and rinsed with distilled water, then germinated on paper towels soaked with distilled water for 2 d. When the roots were about 1.5 cm long, uniform seedlings were transplanted into a growth chamber with a temperature of 22 °C/18 °C, a 14/10 hour light/dark cycle (light for 14 hours from 6 a.m. to 8 p.m.) and 65% humidity. 28-days-old seedlings were transferred to a pre-cooled chamber with a gradually decreasing temperature (1 °C per hour, from 22° to 6°). After cold acclimation, plants were placed at 6° during the day / -5° during the night for 7 days. Throughout the experiment, Water was normally irrigation with the same soil moisture as the water capacity of field. 60 individuals from 6 pots were selected from each material in each period. 12 individuals were used for sequence and physiological analysis. Finally, the survival rate (measured as the percentage of total seedlings that survived in relation to the total number tested) of 16 plants per replicate were measured to evaluate cold tolerance with three replicates. All experiments were performed simultaneously in the light cycle and repeated at least three times.

### Assays of physiological parameters

The total soluble sugar content was calculated according to the standard curve and measured by anthrone colorimetry method with glucose as the standard [85] 0.2 g seedling tissue was ground with 1 mL distilled water, heated for 10 min at 100 °C, cooled, centrifuged at 8000 g for 10 min, and diluted to 10 mL volume with distilled water. The reaction mixture of 0.3 mL includes 0.04 mL extract, 0.04 mL distilled water, 0.02 mL mixed reagent (1 g anthrone and 50 mL ethyl acetate) and 0.2 mL 98% (w / w) H_2_SO_4_. The mixture was heated at 95 °C for 10 minutes, and the absorbance was determined at 620 nm using a Scanning spectrophotometer (Agilent8453e, CA, USA). Malondialdehyde (MDA) content was determined by the thiobarbituric acid reactive substances (TBARS) method using the difference of absorbance at 532 nm and 600 nm [86]. According to the method of WST-8 [87], WST-8 and superoxide anion (O^2^) reaction catalyzed by xanthine oxidase (XO) can produce water-soluble formazan dyes. The activity of superoxide dismutase (SOD) is negatively correlated with the amount of black paint, so the activity of SOD can be calculated by colorimetric analysis of WST-8 products. Peroxidase (POD) enzyme activity can be catalyzed for H_2_O_2_ oxidation of specific substrates by the peroxidase activity detection kit (BC0090, Beijing SolarBio Science & Technology Co., Ltd., Beijing, China), with characteristic light absorption detected at 470 nm [88]. Catalase (CAT) was determined by Li et al. [89] method using catalase kit (S0051, Beyotime Biotechnology, Shanghai, China). According to the method of proline (Pro) content detection kit (BC0295, Beijing SolarBio Science & Technology Co., Ltd., Beijing, China), Pro was extracted with sulfosalicylic acid (SA). Approximately 200 mg seedling tissue was homogenized in 2 mL sulphosalicylic acid (3%), followed by centrifugation at 13000 g for 15 min at 4 °C. The extract (0.5 mL) was transferred to a new microcentrifuge tube and mixed with acid ninhydrin and acetic acid. The reaction mixture was boiled in a water bath at 100 °C for 30 min, cooled at 4 °C for 30 min and thoroughly mixed with 1 mL toluene. Finally, the 0.2 mL absorbance was measured at 520 nm using a spectrophotometer (PowerWave XS2) [90].

### RNA-seq and bioinformatics analysis

As three biological replicas of cold stress treatments and control， twelve fully expanded leaves of 12 individual plants were analyzed by RNA-Seq. Trizol reagents (Invitrogen, CA, USA) was used to extract total RNA. According to the manufacturer’s instructions (Illumina), 100 ng RNA is used to construct the RNA-seq library and synthesize the first-strand cDNA using SuperScript II reverse transcriptase (Invitrogen, CA, USA). After the second-strand cDNA was synthesized and linked, the enriched and purified cDNA fragment was sequenced on the RNA Nano 6000 Assay Kit (Agilent Technologies, CA, USA).

The sequenced raw readings are processed into clean readings by filtering low-quality readings. The clean reads were localized to the reference genome of wheat (http://plants.ensembl.org/Triticum_aestivum/Info/Index) using HISAT2 [91]. The R package, edgeR v3.16, was employed to identify the differentially expressed genes (DEGs) between cold stress and control samples. DEGs of cold-treated plants relative to control samples were detected with a *P* < 0.05 and |log2 ratio ≥ 2|.

as the threshold. Genes were annotated by Nr, Nt, Pfam, KOG/COG, Swiss-Prot, KO, and GO databases. The central and highly connected metabolites of specific modules were identified using Cytoscape v3.9.0 visualizing [92]. Gene ontology (GO) enrichment and Kyoto Encyclopedia of Genes and Genomes (KEGG) pathway enrichment of DEGs were generated using R, based on the hypergeometric distribution. Plant transcription factors were predicted using ITAK software (https://github.com/kentnf/iTAK) [93]. Venny diagrams are drawn using Venny 2.1 on the website (https://bioinfogp.cnb.csic.es/tools/venny/index.html) [94]. Raw and processed RNA-Seq data files were deposited in SRA (https://www.ncbi.nlm.nih.gov/sra/) under the following accession numbers PRJNA800586(https://www.ncbi.nlm.nih.gov/bioproject/PRJNA800586).

### Metabolomic profiling

Twelve fully unfolded leaves from 12 individual plants of three biological repeats were analyzed by metabonomics profiling under stress treatment and control. Each sample (25 mg) was feed into an EP tube containing an internal standard (L-2- Chlorophenylalanine, 2 μg/mL) of 1000 μ L extract solution (acetonitrile: methanol: water = 2:2:1). Samples were incubated (− 40 °C, 1 h) and centrifuged (10,000 rpm, 15 min) after homogenization and sonication cycle. The supernatant (500 μL) was dried in a vacuum concentrator and ultrasonic degradation 200 μL of 50% acetonitrile. 75 μL centrifuged supernatant was transferred to a fresh glass vial for LC/MS analysis. The quality control (QC) sample was made by mixing the supernatants of all samples in equal quantities. Metabolomic analyses were performed using LC-MS as described by Law et al. [95]. Samples were analyzed on an Agilent 7890B gas chromatography system coupled to an Agilent 5977A MSD system (Agilent Technologies Inc., CA, USA). MS raw data files were peak deconvolution, alignment and integration processed by R package XCMS (version 3.2). The metabolome resulting data were identified in MS2 database and analyzed in MetaboAnalyst 4.0 online [96]. The differential metabolites were selected based on the combination of a statistically significant threshold of variable influence on projection (VIP) values obtained from the OPLS-DA model and *p* values from the normalized peak areas from different groups, where metabolites with VIP > 1.0 and *p* < 0.05 were considered as differential metabolites. Hierarchical clustering (Euclidean distance) was performed with MeV4.9 to explore the pattern of metabolite abundances. The ratio of stress-treated plants to control plants was used to calculate the fold-change of each metabolite. As the significance threshold, the variable influence projection (VIP) value of the OPLS-DA model and the *p*-value of two-tailed Student’s t-test on the normalized peak area were used to select the differential metabolites of different groups, in which the metabolites with VIP > 1.0and p < 0.05were regarded as differential metabolites. To explore the distribution of metabolite abundance, MeV4.9 was carried out to analyze hierarchical clustering (Euclidean distance).

### Verification of candidate genes by quantitative real-time PCR (qRT-PCR)

Quantitative real-time PCR (qRT-PCR) was utilized to validate the twelve differentially expressed unigenes transcript expression levels using the same RNA samples used for sequencing. The Specific primers were designed using Primer Premier 6.0 and are listed in Supplementary Table S11. The housekeeping gene was β-actin. According to the user manual, the first cDNA strand fragments were synthesized from total RNA using the PrimeScript™ RT Master Mix kit (Takara, Japan). The qRT-PCR was performed on an ABI7500 real-time fluorescent quantitative PCR instrument (Applied Biosystems, Foster City, USA). Three replicates were performed for each sample. The relative expression levels of the candidate genes were measured using the comparative threshold cycle (2-ΔΔCT) method [41].

### Statistical analysis

All data analyses and graphics drawings were accomplished in R 4.1.2. T The statistical significance was calculated by Student’s t-test. The gene expression values of FPKM were scaled to Z-score to draw the heatmap of transcriptome and metabolome. Graphics and Heatmaps were plotted with the ggplot and the heatmap packages of R language respectively. Venn diagrams were drawn with the Interactivenn website (http://www.interactivenn.net/). Each experiment was repeated three times.

## Supplementary Information

**Additional file 1: Table S1.** Sequencing output statistics of 12 samples. **Table S2.** Detailed information of identified differentially expressed genes. **Table S3.** DEGs identified in all comparison groups. **Table S4.** GO enrichment analysis of the DEGs. **Table S5.** KEGG enrichment analysis of the DEGs. **Table S6.** Significant differences in transcription factors between CK and MU. **Table S7.** Differentially expressed metabolites identified in all comparison groups. **Table S8.** Venn diagram of differentially expressed metabolites were found in all comparison groups. **Table S9.** Significantly differential metabolites and DEGs annotated in phenylpropanoid/flavonoid biosynthesis pathways. **Table S10.** Significantly differential metabolites and DEGs annotated in sugar and amino acids metabolic pathways. **Table S11.** Primers of genes used in RT-qPCR.

## Data Availability

The datasets used and analysed during the current study are available in the NCBI Bioproject repository, [PRJNA800586]. The experimental materials, Jimai 325 and MU-134, were bred and mutated by the Institute of Cereal and Oil Crops, Hebei Academy of Agriculture and Forestry Sciences (HAAFS). Seeds were obtained from the Institute of Cereal and Oil Crops of the Hebei Academy of Agriculture and Forestry Sciences (HAAFS). All databases in this study are available to the public.
